# Physiotherapeutic Approach for Septic Arthritis of Knee Joint

**DOI:** 10.7759/cureus.45550

**Published:** 2023-09-19

**Authors:** Shruti S Bhoge, Subrat Samal

**Affiliations:** 1 Musculoskeletal Physiotherapy, Ravi Nair Physiotherapy College, Datta Meghe Institute of Higher Education and Research, Wardha, IND

**Keywords:** pyogenic arthritis, case report, rehabilitation, knee joint, physiotherapy, septic arthritis

## Abstract

Septic arthritis is an orthopaedic emergency associated with poor prognosis in cases with delayed treatment. The standard routes through which the infection spreads are hematogenous and direct entry. Any delay in medicine could mean the patient facing severe joint destruction, limitation in joint range, and inability to do activities of daily living. Septic arthritis is treated with a multidisciplinary approach in which physiotherapy is essential in making patients functionally independent. This article discusses a 58-year-old male patient with pain and swelling in the right knee joint and difficulty doing activities like walking, squatting and climbing stairs. On further investigations and diagnostic arthroscopy, he was diagnosed with septic/pyogenic arthritis caused by Staphylococcus aureus in the right knee. The patient was being treated with antibiotics. Along with it, patient-tailored physiotherapy rehabilitation, including, but not limited to, strengthening, range of motion (ROM) exercises, endurance training, etc., was also given, which proved highly effective at enhancing the patient's functional independence and quality of life. The outcome measure used in this report is the Knee Injury and Osteoarthritis Outcome Score (KOOS).

## Introduction

Septic arthritis refers to pyogenic bacterial infection of the synovial joint. The knee joint is the most common joint known to be involved, i.e., at least 50% of the cases are related to this joint [1]. At least 2-10 cases per 100,000 people in the general community experience this illness each year. Clinical features like pain, tenderness, swelling, redness, rise in temperature, and decreased range of motion (ROM) can be seen [2]. The most frequent cause of this condition is Staphylococcus aureus, also known to be a cause of non-gonococcal arthritis and can be transmitted distantly from infectious sites via blood or by direct entry via trauma or intra-articular invasive method. Gonococcal arthritis is a type of septic arthritis caused by Neisseria gonorrhoeae in those individuals who are sexually active [1,3]. Joint effusion cartilage leads to increased pressure in the joint, stops the blood flow, and hampers immune reaction, causing damage to articular cartilage. The lysosomal activity of the causative agent on articular cartilage causes further breakdown. In the late stages, there is degeneration of the subchondral bone due to infection [4].

Septic arthritis may result in an extremely bad prognosis if there is any delay in treatment. Therefore, prompt diagnosis with arthroscopy and immediate medical or surgical treatment like arthrotomy washout followed by physiotherapy rehabilitation is crucial for better functional outcomes [5]. The presence can make an early and definite diagnosis of a bacterium responsible for the disease in the synovial fluid [6]. Physiotherapy plays an important role in such a patient's ability to gain functional independence and prevent further complications. Early physiotherapy rehabilitation helps to restore range and strength, decrease pain, and improve functional independence and activity of daily living [7]. This is a case report of a 58-year-old male patient with septic arthritis, and the goal of this paper is to prove the importance of physiotherapy in this condition, which is a common occurrence, yet very few studies have been carried out about it.

## Case presentation

A 58-year-old male, an autorickshaw driver by occupation, who gave an alleged history of slip and fall in a pit 15 days back, had abrasions on the anterior aspect of his knee. Since then, he had mild pain and swelling over his right knee but could walk and do his everyday activities. The patient visited a local hospital where oral painkiller medications were prescribed. His pain suddenly aggravated for one day, associated with diffuse swelling on the anterior aspect of the knee. The pain began slowly and progressed over time. Since then, he had difficulty standing, walking, squatting, and climbing the stairs. He had normal bowel and bladder habits and had no history of associated illnesses. He also gave the history of kharra chewing for 35 years and consumption of alcohol for 30 years.

Clinical findings

On observation, the patient was a mesomorph lying in supine position and was hemodynamically stable. His right knee had three small surgical scars, one on superior lateral aspect of knee and other on inferomedial and inferolateral aspect of knee. After palpating the area, the local temperature was slightly rest and grade 2 tenderness was present. Swelling was also evident. His strength on manual muscle testing (MMT) (according to Medical Research Council Scale) and ROM for bilateral lower limb are mentioned in Tables 1, 2, respectively.

**Table 1:** MMT of lower limbs MMT: Manual muscle testing

Muscles	Right	Left
Hip flexors	4	5
Hip extensors	4	5
Hip abductors	5	5
Hip adductors	5	5
Hip external rotators	5	5
Hip internal rotators	5	5
Knee flexors	2	5
Knee extensors	3	5
Ankle plantarflexors	5	5
Ankle dorsiflexors	5	5
Ankle evertors	5	5
Ankle invertors	5	5

**Table 2:** ROM of hip, knee, and ankle joints ROM: Range of motion

ROM	Right	Left
Hip flexion	0-60˚	0-100˚
Hip extension	0-30˚	0-60˚
Knee flexion	0-10˚	0-128˚
Knee extension	0˚	0˚
Ankle plantarflexion	0-48˚	0-48˚
Ankle dorsiflexion	0-22˚	0-22˚

Radiological findings

**Figure 1:**
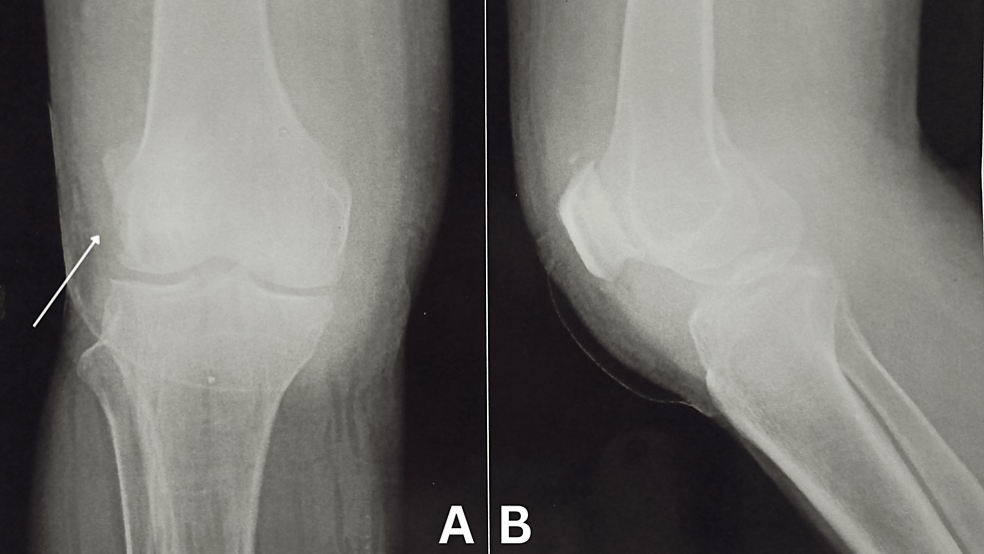
X-Ray of right knee (AP and lateral views) A) AP view; B) Lateral view The arrow points to the sclerotic changes of lateral condyle of femur. AP: Antero-posterior

Ultrasonography of right knee revealed changes of cellulitis in the form of diffuse edema with cobblestone morphology of subcutaneous tissue and increased vascularity on doppler. There was evidence of heterogenous collection in lateral aspect of knee extending into suprapatellar bursa. It also showed evidence of cystic lesion with heterogenous debris between medial heads of gastrocnemius and semimembranosus showing no vascularity n doppler. X-ray showed sclerotic changes of lateral condyle of femur (Figure 1). To confirm diagnosis, the patient underwent diagnostic arthroscopy of right knee where synovium was found to be hypertrophied and articular cartilage was found to be destroyed. Joint fluid was aspirated and synovectomy to shave off excessive synovium was done and both samples were examined further. After microbiological examination of aspirated joint fluid, growth of coagulase negative Staphylococci and coagulase positive Staphylococci were found. Histopathological examination of synovium revealed fibrocollagenous tissue with infiltration of chronic inflammatory cells. All these diagnostic procedures led to the diagnosis of septic arthritis in the right knee.

Timeline

On September 29, 2022, patient was admitted to the orthopaedics ward and after several important investigations and laboratory finding were done patient underwent diagnostic arthroscopy of right knee on October 10, 2022, where joint fluid aspiration and synovectomy was done. The patient was referred for physiotherapy three days after the surgery (Table 3).

**Table 3:** Timeline

Date of admission	September 29, 2022
Date of surgery	October 10, 2022
Date of physiotherapy rehabilitation commencement	October 13, 2022
Date of discharge	October 23, 2022

Therapeutic intervention

Table 4 gives information regarding the phase-wise treatment protocol the patient underwent.

**Table 4:** Physiotherapy intervention SLR: Straight Leg Raise, ROM: Range of Motion; CPM: Continuous passive movement

Phase of rehabilitation	Goals	Intervention and dosage
Phase 1: Early Postoperative Phase (0-6 weeks)	Patient education	A patient's condition, the value of, and the benefits of, physiotherapy are all explained to them. treatment in improving their health condition, avoiding complications and increasing his ability to walk, squat, and other daily activities. The patient is also educated about the hazards of his addictions.
	To maintain muscle strength	Isometric contractions of hamstrings and quadriceps 10 repetitions with 10 sec hold one set. Upper limb strengthening exercises with full 1L water bottle started
	To increase ROM	Relaxed slight passive movement was started to achieve slight knee flexion range. Limb elevation within pain free range (SLR): 10 repetitions 1 set. From postoperative day 6, CPM was given to patient: range 0-30˚ for 15 mins (Figure 2).
	To ensure good ventilation	10 repetitions 2 sets of deep breathing exercises and pursed lip breathing exercises each
	To prevent secondary complications	Ankle pumps: 10 repetitions 2 sets
	To improve gait	Gait training with progressive weight bearing started from postoperative day 4 (Figure 3)
Phase 2: Intermediate Phase (6-12 weeks)	Joint mobility	Full ROM of knee achieved. Active resisted exercises of knee continued
	Muscle strength	Progress to open and closed kinematic chain resistance exercises
	Endurance Training	Low impact aerobic exercises like brisk walking and stationary cycling initiated
Phase 3: Return to Activity Phase (12 weeks and beyond)	Joint mobility and muscle performance	Full active-resisted ROM exercises continued. Progression of resistance for open and closed kinematic chain exercises continued
	Functional retraining and return to activity	Return to full activity by this time period

**Figure 2:**
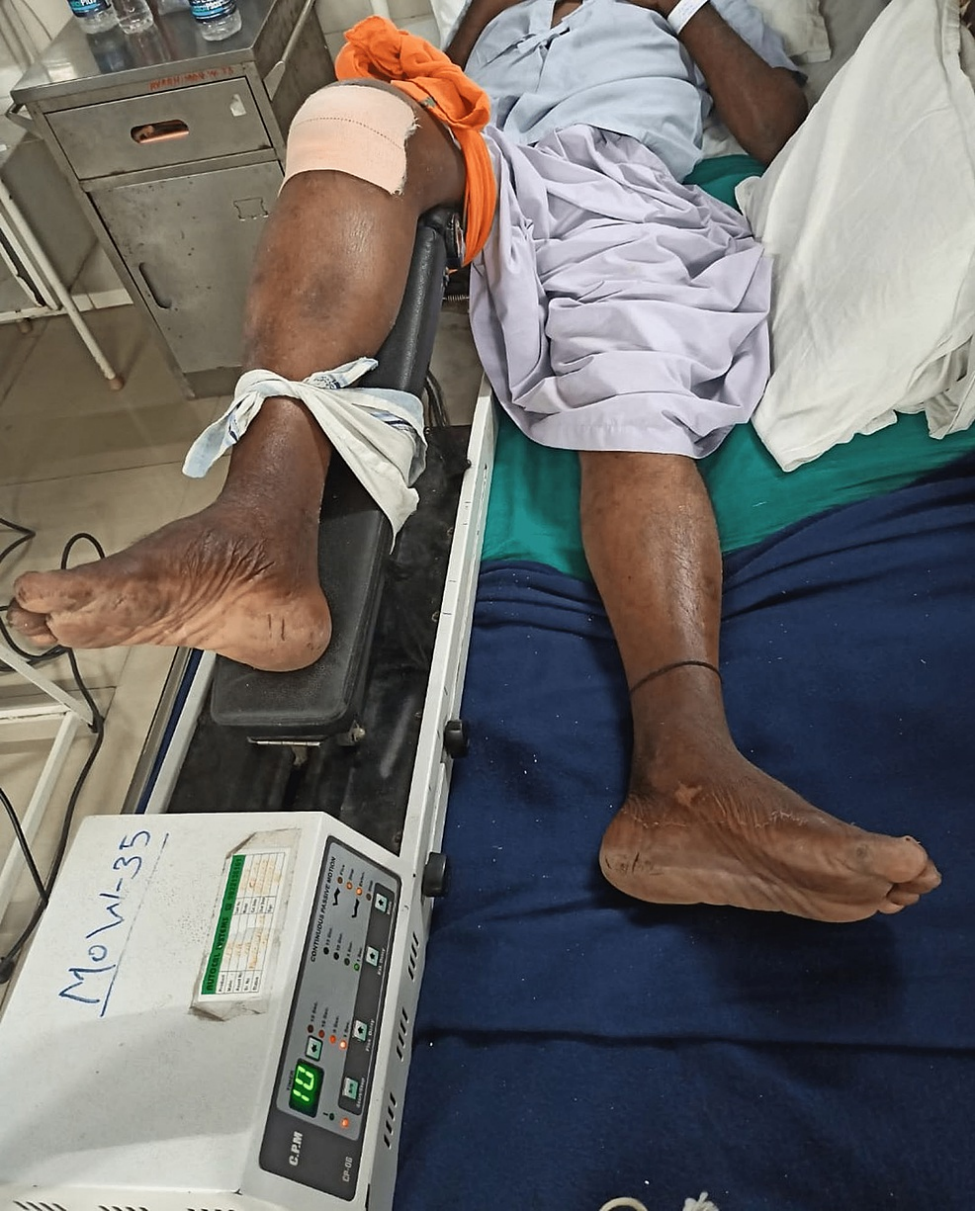
CPM CPM: Continuous passive movement

**Figure 3:**
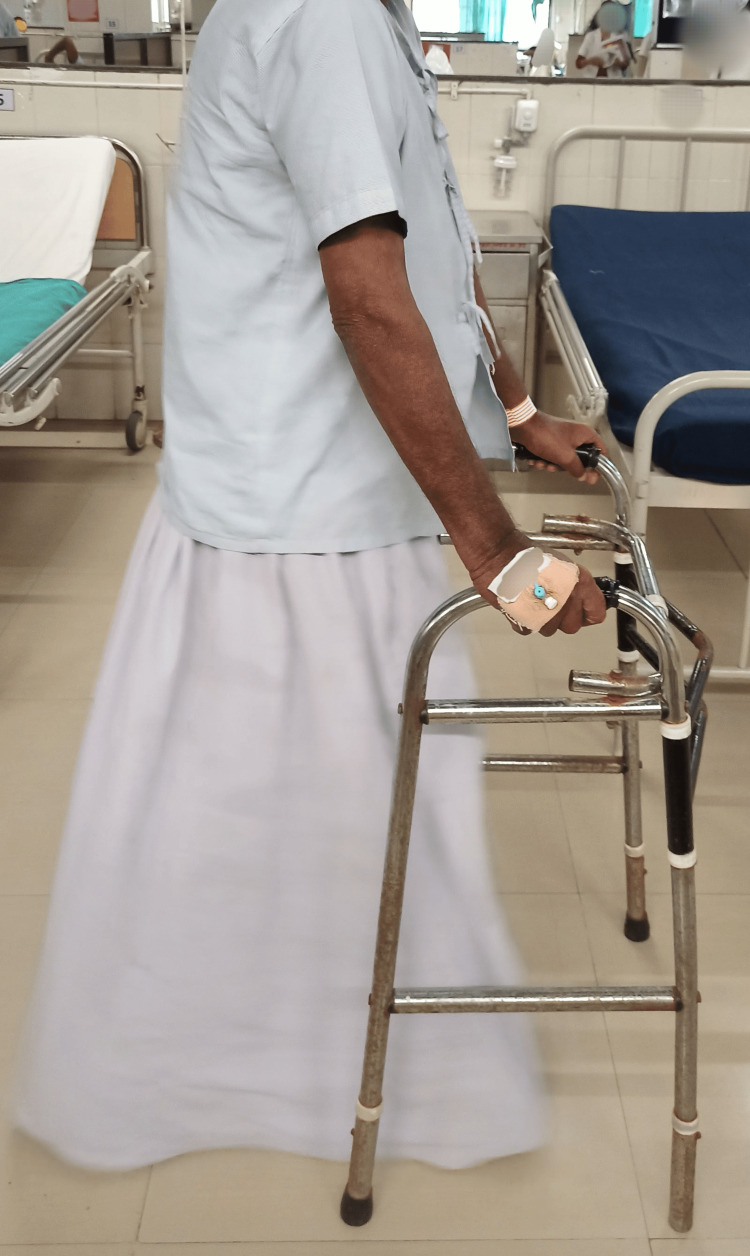
Ambulation

Follow-up and outcome measures

After discharge, the patient came to musculoskeletal physiotherapy for the follow-up for 12 weeks for rehabilitation. At the end of his treatment, assessment was done again and patient reported no complaints of pain and difficulty in doing activities. Functional range was reported. The pre- and postrehabilitation outcomes, including as pain, symptoms, activities of daily living, sport and recreational function, and knee-related quality of life, were assessed using the Knee Injury and Osteoarthritis Outcome Score (KOOS) (Table 5). This scale contains five dimensions with 0 as no problem and 4 as extreme problems. Here, after calculating the score, it is measured on a 0-100 scale, 0 being extreme problem and 100 being no problems.

**Table 5:** Pre- and post-rehabilitation ROM and MMT of right lower limb and KOOS score ROM: Range of motion, MMT: Manual muscle testing, KOOS: Knee Injury and Osteoarthritis Outcome Score

ROM of Right	Pre-rehabilitation	Post-rehabilitation
Hip flexion	0-60˚	0-100˚
Hip extension	0-30˚	0-50˚
Knee flexion	0-10˚	0-120˚
MMT of Right		
Hip flexors	4	5
Hip extensors	4	5
Knee flexors	2	5
Knee extensors	3	5
KOOS Score	48	82

## Discussion

For a positive prognosis and to avoid late complications, septic arthritis is an orthopaedic emergency that requires quick diagnosis and treatment. One of the joints most frequently impacted by septic arthritis is the knee. When the causative agent, i.e., Staphylococcus aureus, enters and adheres to the joint, causing activation of the immune system. This leads to increased permeability of blood vessels in the joint, further leading to leaking of fluid in the interstitium, causing the joint to swell. The synovium and synovial fluid acts as an ideal medium for bacterial growth. As the immune system continues to work, articular cartilage and joint compression are destroyed due to increased intra-articular pressure [2,8].

Wirtz et al. claimed that the best way to treat this condition is by early arthrotomy washout, and debridement is proven to be best for better patient functional outcomes [9]. The patient was given joint mobilisation as early as three days after the surgery, and Couderc et al. also suggested that after surgery, early mobilisation of the joint is recommended to avoid the formation of pannus and to prevent the development of adhesions. Antibiotics are continued until there is no evidence of bacterial growth in synovial fluid [10]. Hamstring and quadriceps strengthening were initiated from the first day of rehabilitation, and Chabaud et al. also recommended improving these muscles first using isometric strengthening, then progressing to dynamic. He also suggested incorporating open-chain exercises in the early rehabilitation and advancing to closed-chain to enhance the strength of these muscles. In this study, these exercises remarkably improved strength [11].

In this report, we saw the use of conventional physiotherapy to improve ROM, strength, quality of life and functional independence. At the end of the rehabilitation program, which lasted for three months, all the treatment outcomes were improved remarkably.

## Conclusions

An emergency condition like septic arthritis needs early diagnosis and prompt treatment to prevent long-term complications. In this case, we used various therapeutic approaches like passive movements, continuous passive movement (CPM) machine, isometric muscle exercises, deep breathing exercises, gait training, and many more, which led to achieving a fully functional range of knee, improved strength, functional independence and improved quality of life in three months after starting physiotherapy rehabilitation. This study provides that even though medical and surgical approaches are important aspects of treating septic arthritis, physiotherapy is just as important as it aids in achieving a person’s functional ability. While in this study, the treatment protocol worked favourably for this patient of septic arthritis, it might not be the case for others, and proper assessment and tailoring the treatment protocol fit to the patient is important.
